# Investigation and analysis of factors related to sleep conditions during the acute withdrawal period of alcohol use disorder

**DOI:** 10.3389/fpsyt.2025.1469324

**Published:** 2025-05-06

**Authors:** Xu Liu, Xiangqi Kong, Xu Chen

**Affiliations:** ^1^ Department of Psychiatry, Shandong Daizhuang Hospital, Jining, China; ^2^ Department of Psychiatry, School of Mental Health, Jining Medical College, Jining, Shandong, China; ^3^ Department Substance Abuse, Shandong Mental Health Center, Shandong University, Jinan, Shandong, China; ^4^ Department of Psychiatry, Jining Medical College, Jining, Shandong, China

**Keywords:** alcohol use disorder, polysomnography, sleep disorder, acute withdrawal, mood disorder

## Abstract

**Objective:**

Patients with Alcohol Use Disorder (AUD) often experience significant mood disturbances and sleep disorders during the acute withdrawal period. This study aims to assess the sleep quality of AUD patients during acute withdrawal using polysomnography (PSG) and to evaluate their emotional states through standardized scales, to explore the role these factors play in the sleep quality of AUD patients during the acute withdrawal period.

**Methods:**

The study’s experimental group consisted of fifty male patients, aged 18 to 66. Fifty healthy male volunteers served as the control group. On days 1–2 of alcohol withdrawal, PSG evaluated sleep processes, structural characteristics, and sleep-related breathing parameters in both AUD patients and the control group. The Pittsburgh Sleep Quality Index (PSQI), Beck Depression Inventory (BDI), Beck Anxiety Inventory (BAI), Penn Alcohol Craving Scale (PACS), and Barratt Impulsiveness Scale (BIS) were used to measure impulsivity, mood disorders, alcohol desire, and sleep quality, respectively. The use of multiple linear regression to analyze factors related to sleep disorders.

**Results:**

Compared to the control group, AUD patients exhibited significantly reduced total sleep time, sleep efficiency, and rapid eye movement (REM) sleep, along with increased snoring frequency and duration. Additionally, AUD patients had significantly higher scores on the PACS, BDI, and BAI. Multiple regression analysis revealed that alcohol craving, depression, and anxiety were significantly associated with impaired sleep quality.

**Conclusion:**

Our findings demonstrate that AUD patients experience significant sleep disturbances during the acute withdrawal period, influenced by alcohol craving, depression, and anxiety.

## 1 Introduction

Alcohol is both a neurotoxin and a central nervous system depressant (1). Excessive intake leads to multi-system damage in the human body (2–5). The core characteristics of AUD include heavy drinking and an inability to control alcohol intake (6), making it a global public health issue responsible for approximately 3 million deaths annually (7). Compared to healthy non-drinkers, AUD patients face more severe sleep and mood problems (8–10). Emotional regulation disorders are key factors driving continued alcohol consumption in AUD patients (11), increasing their risk of relapse when facing negative emotions.

The acute withdrawal period for AUD refers to the hours to days following the last drink (12). During this time, patients may experience symptoms including nausea, tachycardia, hypertension, anxiety, and insomnia (13–15), collectively known as Alcohol Withdrawal Syndrome (AWS). The severity of AWS typically correlates with the degree of alcohol dependence and the duration of alcohol abuse. A systematic review shows symptoms are most severe in the first 2–3 days of acute withdrawal and improve over time (16, 17). We chose to observe patients during the first 1–2 days to better understand the impact of withdrawal on mood and sleep, providing early insights for clinical intervention. Biological processes such as reactive oxygen species (ROS) generation, protein kinase activation, mitochondrial integrity damage, and changes in inflammatory factor levels are critical during alcohol withdrawal (18).

Previous studies have shown that alcohol withdrawal can trigger mood disorders (19), with significant comorbidity between alcohol withdrawal and mood disorders, particularly anxiety and depression. Withdrawal symptoms are closely linked to alcohol craving, emotional disturbances (such as anxiety and depression) (20), and sleep disorders (21), making these factors useful indirect indicators of withdrawal severity. A study on Eastern European populations found a strong association between alcohol withdrawal, drinking, and the occurrence of depressive symptoms (22). Rodent models of alcohol dependence have also demonstrated physical and emotional symptom changes during alcohol withdrawal (23–25). Forced abstinence (FA) in animal models induces anxiety- and depression-like symptoms (26). Six hours after alcohol withdrawal, rats exhibited symptoms such as hyperactivity, agitation, stereotypic behavior, tail stiffness, abnormal gait and posture, and audiogenic seizures (27). However, some studies indicate that depression-like behaviors typically emerge after a longer period of alcohol withdrawal (e.g., 2 weeks) rather than during the acute withdrawal phase. This may be due to the neuroadaptive changes caused by prolonged alcohol consumption, with the central nervous system still recovering during the acute withdrawal phase, leading to unstable emotional states that may later manifest as mood disorders. Sudden cessation of alcohol intake triggers acute central nervous system (CNS) inflammatory responses in key brain regions regulating autonomic and emotional states, affecting mood (28). In alcohol-withdrawn mice, increased expression of kynurenine (KYN), decreased expression of 5-hydroxytryptamine (5-HT), and abnormal expression of 3-hydroxykynurenine (3-HK) and kynurenic acid (KA) were observed in the hippocampus, cerebral cortex, and amygdala, all of which play important roles in mood regulation (29).

Sleep disorders may occur at various stages of AUD, including during long-term drinking and alcohol withdrawal periods (30–33). Heavy drinking can lead to insomnia, and insomnia symptoms may increase the risk of future alcohol consumption (34). These changes are potentially linked to decreased dopamine function and overactivation of stress neuromodulators caused by prolonged heavy drinking (35). A follow-up study of patients abstinent from alcohol for 13 years found a higher incidence of insomnia during the acute withdrawal phase compared to the drinking period (36). Compared to healthy controls, moderate AWS patients exhibit changes in non-rapid eye movement (NREM) sleep, widespread gray matter shrinkage, and cognitive performance decline (37).

Despite the existing literature on alcohol withdrawal and its associated mood and sleep disorders, this study aims to provide further analysis using PSG, combined with patient history, medication use, and sleep laboratory observations, to leverage more objective physiological parameters (38). PSG is recognized as the gold standard for evaluating and diagnosing sleep-related breathing disorders (39). It comprehensively analyzes electroencephalogram, electrooculogram, electromyogram, electrocardiogram, pulse oximetry, airflow, and respiration data to assess the underlying causes of sleep disorders. PSG can monitor arousal, sleep architecture (sleep stage distribution), and arousal frequency (40), and it is also used to assess other sleep disorders such as narcolepsy (41). Studies have found that AUD patients during the acute withdrawal period exhibit sleep abnormalities, including increased sleep latency and fragmentation, reduced sleep duration and efficiency, and a decreased percentage of NREM stage 3 sleep (N3) (42). Reduced slow-wave sleep during this phase, along with limited REM sleep recovery, may be related to the positive allosteric modulation of Gamma-aminobutyric acid type A (GABAA) receptors by alcohol and other mechanisms (43). Reduced REM sleep time has significant implications for mood regulation and sleep quality (44–46). In AUD patients during acute withdrawal, reduced REM sleep may exacerbate mood problems and sleep disorders, potentially creating a negative feedback loop that further affects withdrawal outcomes and long-term recovery. By analyzing the objective differences in sleep quality during the acute withdrawal period in AUD patients, combined with subjective assessments from patient scales, this study aims to further explore the factors influencing sleep during the acute withdrawal period of alcohol use disorder.

## 2 Materials and methods

### 2.1 Study subjects

Patients were screened according to the DSM-5 criteria for AUD, including those diagnosed with alcoholism or alcohol dependence. Due to the requirement for scale assessments and complete PSG monitoring, we selected patients aged 18 to 66 years who could independently complete both the PSG monitoring and scale assessments. The study included male AUD patients newly admitted between December 2021 and December 2022. Out of an initial 287 patients, those with severe neurodevelopmental disorders, neuropsychiatric diseases, or neurological conditions were excluded.

Additionally, patients who had used any antidepressants or antipsychotics in the past two weeks or had a history of other substance dependencies or abuse (excluding tobacco) were also excluded. This was to minimize the potential impact of these substances on the study outcomes. Research has shown that antidepressants and antipsychotic medications can alter mood, sleep architecture, and neurobiological responses, with different drugs having varying effects (47–50). Similarly, other substance dependencies can affect mood and sleep structures (51, 52). Ultimately, 50 eligible male AUD patients were selected for analysis, along with 50 healthy non-drinking males as a control group.

All procedures adhered to the ethical principles outlined in the 1964 Declaration of Helsinki and received approval from the institutional bioethics committee. Informed consent was obtained from all participants.

### 2.2 Sleep and mood assessments

Anxiety symptoms were assessed using the BAI (53), a 21-item self-report questionnaire with scores ranging from 0 to 3 per item, and a total score of 63. Scores above 45 indicate significant anxiety symptoms. Depression severity was assessed using the BDI (54), also a 21-item questionnaire with total scores ranging from 0 to 63. Depression severity is categorized as minimal or no depression (0–4), mild (5–13), moderate (14–20), and severe (21 and above). Impulsiveness was evaluated using the BIS (55), a 30-item questionnaire scored on a scale of 1 to 4, with higher scores indicating greater impulsiveness.

Alcohol craving severity was measured using the PACS (56), which assesses the frequency, intensity, and duration of cravings, the ability to resist cravings, and the average craving level over the past week, on a scale of 0 to 6. Higher scores indicate more severe cravings. Sleep quality was evaluated using the PSQI (57), an 18-item questionnaire covering seven domains: subjective sleep quality, sleep latency, sleep duration, habitual sleep efficiency, sleep disturbances, use of sleeping medication, and daytime dysfunction. Each item is scored from 0 to 3, with a total score of 21. Scores above 7 indicate poor sleep quality. The study group completed these assessments on the 7th-8th day post-enrollment, while the control group completed them on the 1st-2nd day post-enrollment along with sleep monitoring.

### 2.3 Polysomnography monitoring

Data collection and analysis were conducted using the Compumedics Greal PSG 48-channel system. Both the study and control groups underwent PSG assessments on the 1st-2nd day post-enrollment to evaluate sleep processes and structural parameters. Monitored indices included total sleep time (TST), durations of deep and light sleep, REM latency, percentage of REM sleep, apnea-hypopnea index (AHI), number of apnea and hypopnea events, average heart rate, snoring frequency, awakenings, snoring time, average oxygen saturation, and lowest oxygen saturation. These metrics effectively capture changes in sleep structure and physiological baseline data (40).

Both groups underwent consecutive two-night monitoring in a sleep center with controlled ambient conditions (temperature: 18-25°C, relative humidity: 50%-60%). Monitoring occurred from 22:00 to 06:00. Subjects with recordings shorter than 6 hours were re-monitored the next night to eliminate the first-night effect (58). Two professional sleep technicians analyzed the data for all subjects.

### 2.4 Statistical analysis

Data were compiled using Excel 2007 and analyzed with SPSS 26.0. The Shapiro-Wilk and Kolmogorov-Smirnov tests assessed the normality of continuous variables. For normally distributed parameters, independent sample t-tests compared groups, with data presented as mean ± standard deviation. Non-normally distributed parameters were compared using the Mann-Whitney U test, with data presented as median and interquartile range [M (P25, P75)]. Chi-square tests analyzed categorical variables, presented as percentages (rates), with inter-group comparisons using χ² tests. Multiple linear regression analyzed related influencing factors, with statistical significance set at p < 0.05.

### 2.5 Quality control

To ensure the smooth and efficient conduct of the study, rigorous quality control measures were implemented. The project leader provided comprehensive training for researchers on inclusion and exclusion criteria, scale scoring rules, and standardized instructions before the study commenced. All PSG monitoring was conducted in the sleep center, with results analyzed by two professional sleep technicians. Attending physicians evaluated patients for comorbidities or other conditions, excluding those who did not meet the criteria to maintain data integrity. Data entry employed double-entry methods, with 10% of patients randomly selected for consistency checks by two attending or higher-level physicians. Unqualified subjects were excluded to ensure data reliability.

## 3 Results

### 3.1 General characteristics analysis

This study included a total of 100 participants who met the research criteria. The study group comprised 50 males with an age range of 18 to 66 years (mean age: 41.36 ± 11.017 years). The Body Mass Index (BMI) of this group ranged from 17.26 to 35.51 kg/m², with an average of 24.379 ± 4.5078 kg/m². The average years of education were 11.57 ± 2.971 years, with a maximum of 21 years and a minimum of 7 years.

The control group also consisted of 50 males, with an average age of 41.66 ± 9.703 years (range: 26 to 64 years). The BMI in this group ranged from 17.57 to 37.03 kg/m², with an average of 24.4252 ± 3.8636 kg/m². The average years of education in the control group were 12.74 ± 3.28 years, with a maximum of 20 years and a minimum of 7 years.

Statistical analysis revealed no significant differences between the study and control groups in terms of age (*t* = -1.44, *p* = 0.885), BMI (*t* = -0.138, *p* = 0.89), and years of education (*t* = -1.95, *p* = 0.051) (all *p*-values > 0.05) (**Table 1**).

**Table 1:** Comparison of general characteristics between study and control groups.

Variable	Study group(n=50)	Control group(n=50)	t/Z	P
Age(years)	41.36 ± 11.017	41.66 ± 9.703	-1.44	0.885
BMI (kg/m^2^)	24.37 ± 4.5078	24.4252 ± 3.8636	-0.138	0.89
Education(years)	11.57 ± 2.971	12.74 ± 3.275	-1.95	0.051

The general characteristics, including age, BMI, and education level. Values are presented as mean ± standard deviation. The p-values indicate the significance of differences between the study and control groups for each variable.

### 3.2 Comparison of emotional state scores between the two groups

Analysis of the emotional states of the two groups revealed that the study group had significantly higher median scores on the PACS, BDI, and BAI scales compared to the control group. The median (interquartile range) scores for the study group were 12 (8, 17) for PACS, 13.5 (6, 21.25) for BDI, and 31 (24.75, 38) for BAI. In contrast, the control group had median scores of 3.5 (0, 6.25), 6 (3, 11.25), and 22.5 (21, 27) for PACS, BDI, and BAI, respectively. Statistical analysis indicated that the differences in PACS, BDI, and BAI scores between the study and control groups were significant (*t*_PACS_ = -5.672, *p*_PACS_ = 0.000,*t*_BDI_ = -3.516, *p*_BDI_ = 0.000, *t*_BAI_ = -4.952, *p*_BAI_ = 0.000), suggesting that patients in the acute withdrawal phase of AUD experienced more pronounced depression, anxiety, and alcohol craving compared to the healthy control group (*p* < 0.01). However, there was no significant difference in BIS scores between the two groups (*p* > 0.05).

Analysis of sleep quality between the two groups showed that 27 patients (54%) in the study group had a PSQI total score > 7, indicating significant sleep problems, compared to 10 patients (20%) in the control group. Further analysis of the PSQI dimensions revealed that patients in the study group had poorer subjective sleep quality, longer sleep latency, lower sleep efficiency, more sleep disturbances, higher use of sleep medication, and higher total PSQI scores compared to the control group, with statistically significant differences (*p* < 0.05) (**Table 2**).

**Table 2:** Comparison of emotional state and sleep factor scores between study and control groups (M [P25, P75]).

Variable	Study Group (n=50)	Control Group (n=50)	Z	P
PACS	12 (8, 17)	3.5 (0, 6.25)	-5.672	0.000^**^
BDI	13.5 (6, 21.25)	6 (3, 11.25)	-3.516	0.000^**^
BAI	31 (24.75, 38)	22.5 (21, 27)	-4.952	0.000^**^
BIS	53.5 (47, 61.25)	58 (49.75, 63)	-1.394	0.163
PSQI	9 (4, 13)	5 (2.75, 7)	-3.184	0.001^**^
Subjective Sleep Quality	1 (0, 2)	1 (0, 1)	-2.472	0.013^*^
Sleep Latency	1 (1, 2)	0.5 (0, 1)	-3.745	0.000^**^
Sleep Duration	1 (0, 3)	1 (0, 2)	-0.025	0.980
Sleep Efficiency	0 (0, 2)	0 (0, 0)	-3.045	0.002^**^
Sleep Disturbances	1 (1, 2)	1 (0, 1)	-4.526	0.000^**^
Use of Sleep Medication	0 (0, 2)	0 (0, 0)	-3.702	0.000^**^
Daytime Dysfunction	1 (1, 2)	1 (0, 2)	-0.584	0.559

The emotional state was assessed using the PACS, BDI, and BAI scales. Sleep quality evaluation included the PSQI total score and comparisons across its various dimensions. Data are presented as Median (Interquartile Range). ^**^ *p*< 0.01; ^*^ *p*< 0.05.

### 3.3 Analysis of factors affecting sleep quality in AUD patients during acute withdrawal

A multiple linear regression analysis was conducted on the study group, with sleep quality (total PSQI score) as the dependent variable. The independent variables included demographic data (age, BMI, age at first drink, and cumulative drinking duration) and scores from various assessment scales (PACS, BIS, BDI, BAI). This analysis aimed to identify which independent variables significantly impact sleep quality during the acute withdrawal phase in AUD patients.

#### 3.3.1 Influence of general characteristics on sleep quality in AUD patients during acute withdrawal

The multiple linear regression model showed that there were no significant correlations between sleep quality and variables such as age, years of education, age at first drink, cumulative drinking duration, and BMI in AUD patients in acute withdrawal (p > 0.05) (**Table 3**).

**Table 3:** Regression analysis of clinical factors related to sleep quality.

Variable	B	SE	β	T	P
Age(years)	-0.054	0.072	-0.109	-0.742	0.462
Years of Education(years)	0.186	0.27	0.102	0.689	0.494
Age at First Drink(years)	-0.115	0.109	-0.155	-1.06	0.295
BMI(kg/m^2^)	-0.077	0.176	-0.064	-0.435	0.666
Cumulative Drinking Duration(years)	-0.054	0.072	-0.127	-0.742	0.462

In this table, B represents the unstandardized coefficient, SE represents the standard error, β is the standardized coefficient, T is the t-value, and P is the p-value. The analysis indicates that none of the clinical factors considered are significantly related to sleep quality during the acute withdrawal period, as all p-values are greater than 0.05.

#### 3.3.2 Influence of emotional states on sleep quality in AUD patients during acute withdrawal

The multiple linear regression model showed that depression(*b* = 0.153, *t* = 2.68, *p*< 0.05), anxiety(*b* = 0.231, *t* = 3.69, *p* < 0.01), and alcohol craving(*b* = 0.215, *t* = 2.56, *p* < 0.05) were significantly higher in AUD patients during acute withdrawal compared to healthy controls, with all showing statistical significance (*p* < 0.05) (**Table 4**). These findings suggest that alcohol craving, depression, and anxiety may be potential factors contributing to poor sleep quality.

**Table 4:** Regression analysis of emotional factors related to sleep quality.

Variable	B	SE	β	T	P
PACS	-0.215	0.084	-0.309	-2.556	0.014^*^
BDI	0.153	0.057	0.361	2.683	0.01^*^
BAI	0.231	0.063	0.511	3.692	0.001^**^
BIS	-0.025	0.063	-0.043	-0.396	0.694

The emotional factors considered in this analysis include the PACS, BDI, and BAI. The analysis indicates that PACS, BDI, and BAI are significantly related to sleep quality, ^**^ *p*< 0.01; ^*^ *p*< 0.05.

### 3.4 Comparison of various sleep monitoring indicators

#### 3.4.1 Sleep quality analysis

**Figure 1:**
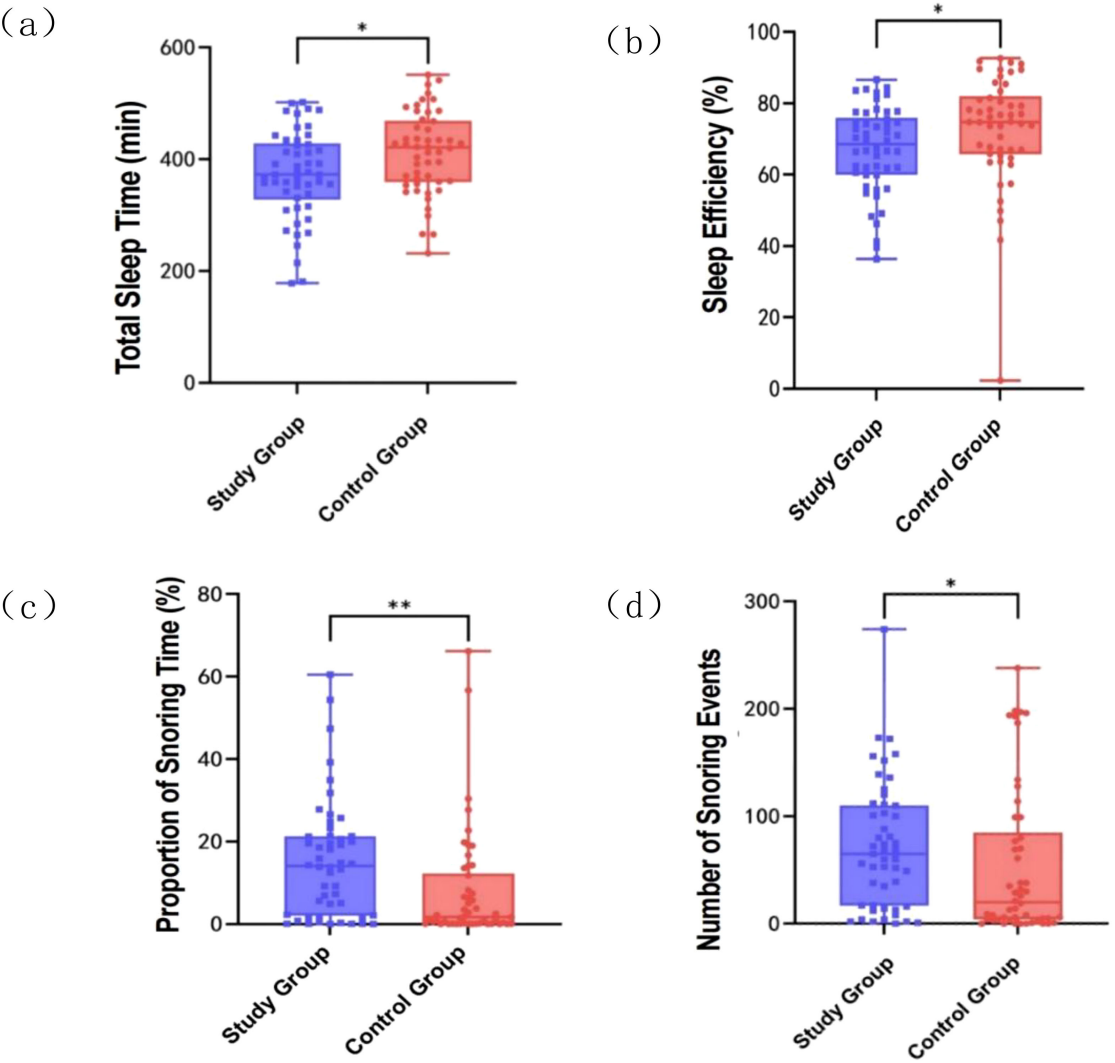
Comparison between the study group and the control group in various sleep quality indicators. **(a)** Comparison of the distribution of total sleep time in the study and control groups; **(b)** Comparison of sleep efficiency between study and control groups; **(c)** Comparison of proportion of snoring time between study and control groups; **(d)** Comparison of number of snoring events between study and control groups. ^**^ *p* < 0.01; ^*^ *p* < 0.05.

Analysis of sleep quality between the two groups showed no significant difference in sleep onset latency (*t* = -1.037, *p* = 0.302) or the number of awakenings (*t* = -1.722, *p* = 0.088). However, compared to the control group, the study group exhibited significantly reduced sleep efficiency (*t* = 2.161, *p* = 0.033), decreased total sleep time (*t* = 2.612, *p* = 0.01) (**Figure 1a**), poorer sleep efficiency (*t* = 2.161, *p* = 0.033) (**Figure 1b**), increased proportion of snoring time (*t* = -3.44, *p* = 0.001) (**Figure 1c**), and a higher number of snoring events (t = -2.3, *p* = 0.021) (**Figure 1d**).

#### 3.4.2 Sleep architecture analysis

The analysis of sleep architecture between the two groups showed a significant difference in the duration of REM sleep (*t* = 2.924, *p* = 0.004), with the study group having significantly less REM sleep compared to the control group.

When analyzing the different sleep stages, no significant differences were found in the duration of deep sleep (*t* = 1.729, *p* = 0.087), light sleep (*t* = 0.555, *p* = 0.58), or the proportions of these stages in total sleep time, including deep sleep percentage (t = 1.136, *p* = 0.259), light sleep percentage (*t* = -1.402, *p* = 0.164), and REM sleep percentage (*t* = 1.523, *p* = 0.131) (**Table 5**).

**Table 5:** Comparison of sleep architecture indicators ( $\bar{x}$ ± *s*), M (P25, P75).

Indicator	Study Group	Control Group	T	P
Duration of Each Stage (min)				
Deep Sleep	55.58 ± 51.243	75.12 ± 61.342	1.729	0.087
Light Sleep	232.22 ± 69.767	239.7 ± 64.834	0.555	0.58
REM Sleep	84.08 ± 21.372	96.48 ± 21.04	2.924	0.004^*^
Deep Sleep (%)	14.838 ± 13.2338	17.848 ± 13.2624	1.136	0.259
Light Sleep (%)	62.602 ± 13.1839	58.892 ± 13.2689	-1.402	0.164
REM Sleep (%)	22.558 ± 2.8187	23.478 ± 3.2105	1.523	0.131

For parameters that follow a normal distribution, we used an independent samples t-test for between-group comparisons, and data are presented as mean ± standard deviation. ^*^ *p*< 0.05.

#### 3.4.3 Analysis of AHI index and general vital signs during sleep

The AHI index refers to the average number of apnea and hypopnea events per hour of sleep, indicating the presence of sleep apnea and hypopnea.

Analysis of the AHI index between the two groups showed no significant differences in the average number of apnea and hypopnea events (*t* = -0.172, *p* = 0.863), suggesting no difference in sleep quality due to breathing problems between the groups.

Furthermore, analysis of general vital signs during sleep indicated no significant differences in the number of hypopnea events (*t* = -0.093, *p* = 0.926), number of apnea events (*t* = -0.345, *p* = 0.73), average heart rate (*t* = -0.77, *p* = 0.443), average oxygen saturation (*t* = -1.105, *p* = 0.272), or lowest oxygen saturation (*t* = -0.343, *p* = 0.733) between the two groups, suggesting that differences in general vital signs do not account for sleep quality differences (**Table 6**).

**Table 6:** Comparison of general vital signs during sleep ( $\bar{x}$ ± *s*), M (P25, P75).

Indicator	Study Group	Control Group	t/Z	P
AHI	2.9 (0.8, 7.45)	2.25 (1.075, 4.5)	-0.172	0.863
Number of Hypopnea Events	15 (4,30.25)	14.5 (7,23.25)	-0.093	0.926
Number of Apnea Events	1 (0,3)	1 (0,4.25)	-0.345	0.73
Average Heart Rate	70.33 ± 10.003	68.708 ± 11.0486	-0.77	0.443
Average Oxygen Saturation	95.76 ± 1.333	93.76 ± 12.734	-1.105	0.272
Lowest Oxygen Saturation	84.16 ± 8.681	83.52 ± 9.953	-0.343	0.733

Data are presented as mean ± standard deviation ( $\bar{x}$ ± s) for normally distributed variables, and as median (M) with interquartile range (P25, P75) for non-normally distributed variables. No significant correlations (p>0.05) between sleep quality and the general characteristics listed.

### 3.5 Correlation analysis of clinical psychological symptoms and sleep quality

#### 3.5.1 Correlation between clinical psychological symptoms and sleep quality in the study group

Analysis of the quantified data of clinical psychological symptoms and sleep quality showed no significant correlation between sleep onset latency, total sleep time, sleep efficiency, and the number of awakenings with the total scores of emotional scales. However, there was a positive correlation between snoring time and PACS and BDI (*r* = 0.359, *p* < 0.05; *r* = 0.281, *p* < 0.05), and between the number of snoring events and BDI (*r* = 0.359, *p* < 0.01) (**Table 7**).

**Table 7:** Correlation analysis between clinical psychological symptoms and sleep quality.

Variable	Sleep Onset Latency	Total Sleep Time	Sleep Efficiency	Snoring Time	Number of Snoring Events	Number of Awakenings
PACS	0.127	-0.009	0.029	0.359*	0.267	-0.151
BDI	-0.118	0.115	0.195	0.281*	0.396**	-0.149
BAI	-0.018	0.16	0.265	0.187	0.245	-0.179
BIS	0.035	0.03	0.026	0.086	0.219	-0.049

Correlations between clinical psychological symptoms (PACS, BDI, BAI, BIS) and various sleep quality measures are presented. Values represent correlation coefficients. Positive values indicate a direct relationship, while negative values indicate an inverse relationship. Significance levels are indicated as follows: ^*^ *p* < 0.05; ^**^ *p* < 0.01.

#### 3.5.2 Correlation between clinical psychological symptoms and sleep architecture in the study group

Analysis of the quantified data of clinical psychological symptoms and sleep architecture showed no significant correlation between light sleep, REM sleep, and the proportions of these stages with the total scores of emotional scales. However, there was a positive correlation between BAI and the duration of deep sleep (*r* = 0.310, *p* < 0.05), as well as the proportion of deep sleep (*r* = 0.305, p < 0.05) (**Table 8**).

**Table 8:** Correlation analysis between clinical psychological symptoms and sleep architecture.

Indicator	Deep Sleep	Light Sleep	REM Sleep	Deep Sleep %	Light Sleep %	REM Sleep %
PACS	0.246	-0.176	-0.05	0.231	-0.207	-0.113
BDI	0.233	-0.064	0.075	0.26	-0.246	-0.072
BAI	0.310^*^	-0.093	0.154	0.305^*^	-0.206	0.001
BIS	-0.092	0.096	0.017	-0.056	0.051	0.025

Correlations between clinical psychological symptoms (PACS, BDI, BAI, BIS) and various aspects of sleep architecture are shown. Values represent correlation coefficients. Positive values indicate a direct relationship, while negative values indicate an inverse relationship. Significance levels are marked as follows: *p < 0.05.

#### 3.5.3 Correlation between clinical psychological symptoms and AHI index and general vital signs in the study group

Analysis of the quantified data of clinical psychological symptoms, AHI index, and general vital signs showed no significant correlation between AHI, the number of hypopnea events, the number of apnea events, average oxygen saturation, and lowest oxygen saturation with the total scores of emotional scales. However, there was a positive correlation between BAI and average heart rate (*r* = 0.369, *p* < 0.01) (**Table 9**).

**Table 9:** Correlation analysis between clinical psychological symptoms and sleep process data.

Variable	AHI	Number of Hypopnea Events	Number of Apnea Events	Average Heart Rate	Average Oxygen Saturation	Lowest Oxygen Saturation
PACS	-0.134	-0.175	-0.066	0.206	-0.258	-0.062
BDI	-0.015	0.051	-0.11	0.13	0.013	0.076
BAI	-0.045	0.01	-0.121	0.369^**^	-0.027	0.086
BIS	0.084	0.122	0.001	0.026	0.062	-0.077

Correlations between clinical psychological symptoms (PACS, BDI, BAI, BIS) and various sleep process data are presented. Values represent correlation coefficients. Positive values indicate a direct relationship, while negative values indicate an inverse relationship. Significance levels are marked as follows: **p< 0.01.

## 4 Discussion

This study reveals significant issues in anxiety, depression, alcohol craving, and sleep disturbances among patients with AUD during acute withdrawal. Compared to non-drinkers, these patients exhibit markedly poorer sleep quality, sleep architecture, and related physiological indicators. Specifically, the total sleep time is significantly reduced, REM sleep duration and proportion are lower, and overall sleep efficiency is decreased in AUD patients. Our findings indicate a strong correlation between sleep disturbances and the severity of anxiety, depression, and alcohol craving in these patients—greater symptom severity is associated with poorer sleep quality.

Consistent with previous studies, we found that AUD patients in the acute withdrawal phase often experience significant anxiety and depression (59–61). These emotional disturbances are closely linked to the level of alcohol dependence during withdrawal (62). Importantly, these emotional issues and cravings for alcohol are key drivers of relapse in AUD patients (63). During acute withdrawal, relapse may alleviate emotional problems by modulating brain neurotransmitters (64). Chronic heavy drinking is known to cause emotional and cognitive disorders (65), and alcohol-related brain damage accounts for 10% of mild neurocognitive disorders (66). Although we observed emotional disturbances in alcohol withdrawal patients, further research is needed to understand the specific impact of drinking on emotions and to track the changes in emotional disturbances from drinking to withdrawal dynamically. Future studies should focus on the drinking population to explore the complex relationship between alcohol consumption and emotional disorders.

Using the PSQI, we found significant sleep disturbances among AUD patients in the acute withdrawal phase. This finding aligns with numerous previous studies showing widespread sleep problems during acute alcohol withdrawal (67, 68). However, the PSQI, being a subjective tool, has limitations. To objectively evaluate sleep conditions in AUD patients during acute withdrawal (69), we utilized PSG to analyze various physiological parameters and examine the relationship between specific sleep abnormalities and emotional problems. Compared to healthy controls, AUD patients in the acute withdrawal phase exhibited significantly more snoring time and frequency, which correlated with emotional disturbances. Previous studies have indicated that snoring during sleep increases the risk of depressive symptoms (70), and maternal snoring has been identified as a risk factor for prenatal depression (71). Notably, emotions also affect sleep (72). These findings support our observation that a bidirectional relationship may exist between sleep disturbances and mood disorders in AUD patients. However, research on the interaction between sleep and mood disorders in AUD populations remains relatively limited, highlighting the necessity for future studies in this field. Particularly noteworthy is that in-depth investigation into how sleep disturbances affect emotional states in AUD patients, and whether sleep improvement can effectively alleviate emotional problems in these patients, will hold significant clinical importance.

We also found significant differences in sleep architecture between AUD patients in the acute withdrawal phase and healthy controls, particularly in reduced REM sleep duration and proportion. Previous research has shown shortened REM latency and reduced REM sleep percentage during acute withdrawal. Our study aligns with George F. Koob et al.’s review (35), indicating that REM sleep decreases and recovery is limited during the acute withdrawal phase in AUD patients. REM sleep’s macro and microstructure are crucial for learning and memory consolidation (73), and alcohol withdrawal primarily affects the prefrontal cortex, striatum, and hippocampus functions, leading to cognitive dysfunction (74). Thus, reduced REM sleep during acute withdrawal may relate to persistent cognitive impairment and relapse risk.

It is important to note that AUD patients experience sleep problems during both heavy drinking and early remission, as well as during acute withdrawal (30, 75), mainly manifested as prolonged sleep latency and altered sleep structure (76). Analyzing sleep abnormalities in AUD patients during acute withdrawal suggests that these issues result from multiple factors (77), including physiological, psychological, and environmental influences.

Insomnia and emotional dysregulation in AUD patients are closely related to anxiety, post-traumatic stress disorder, and the severity of drinking (78). Emotional dysregulation and sleep disturbances have a complex bidirectional relationship. Anxiety and depression activate the HPA axis, which exacerbates sleep disorders, and the HPA axis itself is disrupted during alcohol withdrawal, leading to issues such as nighttime awakenings and prolonged sleep latency (79, 80). Poor sleep quality, in turn, increases stress and reduces emotional regulation, further worsening mood disorders (81). Additionally, alcohol withdrawal alters the central nervous system’s neurotransmitter systems, reducing inhibitory effects and increasing excitatory activity, which intensifies symptoms such as anxiety, depression, and sleep disturbances (82–85).

During withdrawal, increased alcohol cravings and accompanying depressive and anxious emotions lead to more pronounced sleep disturbances. The simultaneous presence of anxiety and emotional dysregulation may increase the risk of insomnia, while insomnia, in turn, may exacerbate emotional dysregulation and anxiety, though not all sleep disturbances are directly related to anxiety. Our study shows that AUD patients in the acute withdrawal phase have higher alcohol cravings and more pronounced depressive and anxious emotions, leading to more severe sleep disturbances. Insomnia itself can cause emotional dysregulation and anxiety, forming a self-perpetuating cycle. While not all sleep disturbances are directly linked to anxiety (86), we believe that alcohol withdrawal is a key initial factor causing sleep disturbances. Emotional issues (such as anxiety and depression) further negatively impact sleep quality, influenced by previous drinking levels and alcohol cravings. During withdrawal, alcohol-related sleep disturbances exacerbate overall sleep problems. This interaction suggests a complex relationship between alcohol withdrawal, emotional problems, and sleep disturbances.

This study underscores the critical interplay between emotional dysregulation, elevated craving, and impaired sleep architecture during acute alcohol withdrawal. Notably, reduced REM sleep emerges as a potential relapse predictor, emphasizing the therapeutic imperative of sleep restoration. PSG provides objective biomarkers to guide personalized interventions. Understanding these associations is crucial for developing effective clinical intervention strategies, particularly by integrating emotional regulation and sleep quality treatments to improve patient symptoms more comprehensively.

Our in-depth analysis recognizes several limitations. Firstly, although the study focuses on sleep problems and emotional disturbances during acute alcohol withdrawal, existing research has found a complex relationship between drinking behavior, sleep duration, and depressive symptoms (87). Evidence shows that AUD patients also experience these issues while drinking, which may worsen during withdrawal; however, we did not thoroughly explore the patients’ conditions during drinking. Moving forward, we should further investigate these issues during alcohol use to gain a deeper understanding of the relationship between alcohol consumption, sleep, and mood. Secondly, the study mainly assesses patients during the acute withdrawal phase without dynamically tracking sleep and emotional changes throughout the withdrawal process. Additionally, gender differences may play a significant role in the course of AUD, symptom presentation, and physiological and psychological responses during withdrawal (32), but this study only includes male patients, lacking data on female patients. Furthermore, the bidirectional relationship between insomnia, anxiety, and emotional dysregulation complicates constructing a comprehensive model to explain these interactions. The patients in the study quit smoking during hospitalization, while the complex relationship between smoking and sleep and emotional states (70) might confound withdrawal symptoms like anxiety, irritability, and sleep disturbances with alcohol withdrawal symptoms. We also acknowledge that certain personality factors (such as extraversion and openness) may have a modest influence on sleep quality (88). Additionally, low socioeconomic status (SES) has been linked to poor sleep quality, although the underlying mechanisms remain unclear (89). Although we used PSG for objective assessment, the limited sample size and short monitoring period may not fully capture long-term sleep problems. Finally, despite efforts to control environmental factors, the hospital environment itself, with fixed schedules, medical monitoring, and relative quietness, might impact patients’ sleep and emotions. Our research employed objective measures, such as PSG to systematically analyze sleep parameters during alcohol withdrawal, particularly changes in REM sleep, providing more precise data. Furthermore, our study highlighted the bidirectional relationship between sleep and emotional disturbances, a complex interaction that has not been thoroughly explored in the existing literature. Future research needs to more comprehensively understand and address sleep disturbances and emotional dysregulation in AUD patients, providing more effective clinical guidance and intervention strategies. The Charlson Comorbidity Index (CCI) is often used to assess comorbidities (90). While we excluded patients with major psychiatric disorders and substance dependence, conditions like liver disease or diabetes may still impact mood and sleep during alcohol withdrawal. Future studies should use the CCI and analyze the effects of comorbidities on mood and sleep in these patients.

## 5 Conclusion

Our study reveals patients with alcohol use disorder (AUD) in the acute withdrawal phase exhibit significant emotional disturbances and sleep problems. PSG is an effective tool for identifying these sleep issues in patients. Further analysis indicates a strong correlation between sleep quality and levels of depression, anxiety, and alcohol craving. Regular evaluation of sleep problems from both subjective and objective perspectives is beneficial in the treatment of AUD patients. Additionally, addressing emotional disturbances, anxiety, and sleep issues concurrently may enhance treatment outcomes for patients in the acute withdrawal phase, as these problems often interact and exacerbate each other. Therefore, an integrated approach that considers these factors to improve treatment efficacy is recommended.

## Data Availability

The original contributions presented in the study are included in the article/Supplementary Material. Further inquiries can be directed to the corresponding author.
